# Expression of Tumour-Associated MUC1 Is a Poor Prognostic Marker in Breast Cancer in Kumasi, Ghana

**DOI:** 10.1155/2020/9752952

**Published:** 2020-04-22

**Authors:** E. Atta Manu, K. Bedu-Addo, N. A. Titiloye, C. Ameh-Mensah, F. Opoku, B. M. Duduyemi

**Affiliations:** ^1^Department of Physiology, Kwame Nkrumah University of Science and Technology, Kumasi, Ghana; ^2^Department of Pathology, Kwame Nkrumah University of Science and Technology, Kumasi, Ghana

## Abstract

**Background:**

Immunohistochemical assessment of breast cancer and stratification into the basic molecular subtypes afford a much deeper insight into the biology of breast cancer, while presenting with opportunities to exploit personalized, targeted treatment. Traditionally, the oestrogen, progesterone, and epidermal growth factor receptors are assessed. MUC1, a transmembrane mucin, has been demonstrated a potential prognostic and metastatic marker in breast cancer. However, there have been a limited number of studies addressing the predictive and prognostic features of MUC1 in African breast cancer. This study aims at addressing the expression profiles of MUC1 and other biomarkers in Ghanaian breast cancer and determines its predictive and prognostic characteristics, in relation to other clinicopathological features.

**Methods:**

Haematoxylin and eosin (H&E) slides of breast cancer cases were reviewed and 203 suitable cases were selected for tissue microarray (TMA) construction and immunohistochemistry. Anti-ER, PR, HER2, Ki-67, and MUC1 antibodies were used. Results from the immunostaining were analysed using SPSS version 23.

**Results:**

About 59% of cases expressed MUC1. Majority of cases in the study showed a lack of expression of all three traditional markers (29% expressed ER, 10.9% PR, and 20.7% HER2). Ki-67 index were 62.1% (low), 16.5% (moderate), and 21.4% (high). MUC1 expressions among the molecular classes were luminal A (60.7%), luminal B (68.8%), HER2 overexpression (87.5%), and triple negative (56.6%). There were significant associations between MUC1 and HER2 overexpression (*p*=0.01) and triple negative (*p* < 0.01).

**Conclusion:**

The high proportion of breast cancer cases expressing MUC1, as well as its association with the two most aggressive molecular classes, indicate a substantial role in the biology of breast cancer in our cohort, and it is an indication of poor prognosis.

## 1. Introduction

Breast cancer, the most commonly diagnosed cancer type in women globally, has remained an important health challenge for decades. With an estimate of 2 million newly diagnosed cases and a corresponding 626,679 deaths in the year 2018, breast cancer has proven to be a major barrier to improvements in life expectancy worldwide [1].

Appreciable improvements have been made in the diagnosis, treatment, or management of breast cancer, especially in the developed countries [2–4]. The frequent occurrence of hormone-positive breast cancers among whites in these developed countries [5–7], and the administration of targeted therapies that antagonize the activity of oestrogen and/or progesterone such as tamoxifen after adjuvant chemotherapy, has led to great reductions in the breast cancer-specific mortality rates in these countries [8].

Additionally, the use of trastuzumab, pertuzumab, and other therapies for HER2+ breast cancer has contributed immensely towards an improvement in the overall survival of breast cancer patients [9–11]. Consequently, percentages of 5-year survival with breast cancer are documented to be over 80% in the United States and Europe [4, 12, 13].

In Africa, however, alarming increase in the incidence of breast cancer [1], which is mostly of aggressive histological characteristics and frequent lymph node metastasis, presents a major health challenge to women. This challenge is compounded by issues associated with access to healthcare, diagnosis, treatment, and management of the disease, especially in low-resource settings [2, 14, 15].

Central to the challenges faced in the treatment and management of breast cancer in Africa is the fact that African breast cancer exhibits distinct molecular characteristics from that presented by Caucasians [16]. Although variations in frequencies have been reported across the continent, African women are known to present with the highest proportions of receptor-negative or triple-negative breast cancers [2, 14, 17]. This indicates that a substantial proportion of African women diagnosed of breast cancer are unable to benefit from anti-ER nor anti-HER2 adjuvant therapies and, in the absence of alternative molecular targets, must resort to conventional chemotherapy. In line with the assertion that triple-negative breast cancers are a heterogenous group, there are variable responses to administered chemotherapy, and the outcome for a number of cases are still unfavourable [18, 19].

Consequently, survival among African women with breast cancer is much lower compared to that of Whites, with as low as 13.6% in Gambia [20]. In line with the lower rates particularly in sub-Saharan Africa, a recent study on survival outcomes of breast cancer in Ghana demonstrated that the overall 5-year survival was 47.9% [21]. This underscores the need for further biomarker research to identify predictive/prognostic markers which may be amenable for improved treatment.

MUC1, a single-pass, type 1 transmembrane glycoprotein that is expressed only on the apical surfaces of normal glandular and ductal epithelial cells [22, 23], has been shown to be a potential target for cancer therapy, ranking as the 2^nd^ cancer vaccine target among 75 other cancer antigens assessed by the National Cancer Institute [24]. This rank was based on the tumour-specific aberrant glycosylation pattern of MUC1 in various forms of adenocarcinoma. The hypoglycosylated MUC1 is overexpressed and is expressed on the whole cell surface, in contrast to the apical expression on normal cells [25]. Because of the aberrant glycosylation, T- and B-cell epitopes in the peptide backbone are now accessible, forming tumour-associated MUC1 antigens [23, 26]. Accordingly, a number of clinical and preclinical studies have delved into MUC1-mediated cancer immunotherapy and vaccination (reviewed elsewhere [27–29]).

Expectantly, studies have been designed to assess the predictive and prognostic importance of MUC1 in various forms of cancer [30], including invasive and metastatic breast cancer [31–33], with the aim of establishing a basis for improved diagnosis and the potential benefits MUC1 immunotherapy could afford. Although MUC1 is overexpressed in the majority of breast cancer cases studied [13, 32–36], there appears to be racial differences in the proportion of positives and associated prognosis. Despite this variation, studies of MUC1 in African breast cancer have been sparse, and currently, no single such study has been done here in Ghana.

This study is designed to assess the proportion of MUC1 positives and establish the predictive and prognostic significance of MUC1 in primary breast carcinoma presented by Ghanaian women in Kumasi.

## 2. Materials and Methods

### 2.1. Ethical Consideration and Study Samples

Following approval (REG NO : RD/CR18/203) from the Research and Development Unit, Komfo Anokye Teaching Hospital (KATH), ethical approval (Ref : CHREP/AP/417/18) was obtained from the Committee on Human Research Ethics and Publication, Kwame Nkrumah University of Science and Technology (KNUST), under the title “Molecular Profiling of Breast Cancer in Kumasi” prior to execution of the study protocol.

The study population comprised consecutive cases of female patients visiting the breast clinic at KATH from 2009 to 2017, with primary breast cancer tissues preserved as formalin-fixed paraffin embedded (FFPE) blocks. Patient demographics were abstracted from a database at the Department of Pathology, KATH, where the samples had been sent for histopathological appraisal. The information included age, sex, histological diagnosis, tumour grade, and lymphovascular invasion.

Haematoxylin and eosin- (H&E-) stained slides were made from the FFPE blocks for joint review by two pathologists (NAT and BDM). Review was made according to guidelines outlined by the WHO [37]. Clinicopathological data were confirmed or amended where applicable. The most representative tumour areas were marked on the H&E slides to aid in the construction of a tissue microarray (TMA).

### 2.2. Tissue Microarray (TMA) Construction

TMA was constructed with slight modifications to the procedure described previously [38]. Briefly, recipient paraffin blocks were constructed using a silicone mould supplied with a manual TMA machine (Micatu MicaArray Gen. 4). Following the delineation of a TMA map according to the case identification numbers, the FFPE tissue blocks (henceforth referred to as “donor blocks”) were oriented and the representative tumour foci identified with the aid of the reviewed H&E slides. Successively, two cylindrical cores (diameter: 1.0 mm each) of tissue were extracted from each donor block and inserted into predrilled holes in the TMA recipient block. After insertion of the tissue cores, the recipient block was heated gently under an incandescent lamp to allow the tissue cores to sink in further.

### 2.3. Immunohistochemistry (IHC)

Immunohistochemical staining was performed according to standard procedures. Antibodies for cyclin D1, estrogen receptor (ER), HER2, Ki-67, MUC1, and progesterone receptor (PR) were used. 3 *µ*m thick sections were made from each TMA block onto SuperFrosted Plus slides and taken through deparaffinization using xylene. The sections were subsequently rehydrated using a series of ethanol solutions of decreasing grades (absolute-95%–70%), diluted with tris buffered saline (TBS), and washed with distilled water. Antigen retrieval was performed by incubating the TMA sections in citrate buffer (pH 6) in a pressure cooker (10 min). Background staining and nonspecific antibody binding were prevented using hydrogen peroxide (3%) in methanol for 10 minutes and casein solutions, respectively. The sections were incubated with the primary antibodies according to the manufacturers' specifications (shown in Table 1). Secondary antibody conjugated with peroxidase and anti-peroxidase (DAKO) was added. Sections were developed later with diaminobenzidine tetrahydrochloride (DAB). The sections were counterstained in haematoxylin, dehydrated in increasing grades of ethanol (70%-95%-absolute), and mounted using the DPX mountant.

### 2.4. Scoring of IHC

IHC-stained sections were scored according to percentages of cells positive for the various biomarkers.

### 2.5. Statistical Analysis

Records on patient demographics, clinicopathological characteristics, and results from IHC were analysed using the Statistical Package for Social Sciences (SPSS) version 23. Associations in expression of biomarkers and clinicopathological characteristics and demographics were explored using the chi-squared test. A *p*-value ≤ 0.05 at the 95% confidence level was considered statistically significant.

## 3. Results

A total of 203 cases had preserved blocks with representative tumour foci for the study period, hence formed the study samples. Descriptive statistics of the cases' demographics and histological characteristics are detailed in Table 2. The mean age of the cases was 49.34 years, and invasive carcinoma NST was the predominant histological type (83.0%). Majority of the cases were of histological grade III (55.7%), while 50.8% were negative for lymphovascular invasion.

### 3.1. Immunohistochemical Characteristics

**Figure 1:**
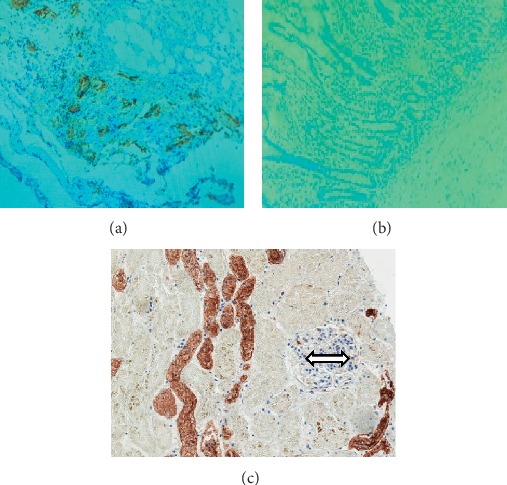
(a) Positive control (colonic tumour), (b) negative control (healthy colon tissue), and (c) negative internal control (breast cancer (nontumour, closed arrow)) for tumour-associated MUC1. The golden brown colours signify positive results.

**Figure 2:**
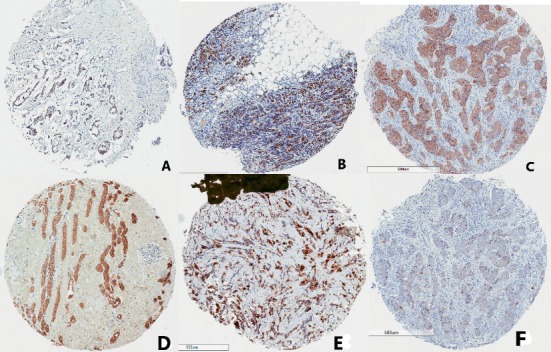
Photomicrographs of cases positive for ER (A), PR (B), HER2 (C), MUC1 (D), cyclin D (E), and high Ki-67 (F) immunohistochemistry. The golden brown colours signify positive tumour areas.

Majority of the cases were negative for expression of ER, PR, and HER2. The respective negative percentages are 71.0, 89.1, and 79.3. MUC 1 was rather overexpressed in majority of cases, with a percentage of 59.0.  Table 3 details the immunohistochemistry results of the biomarkers, along with Ki-67, for which 62.1% of cases had a low expression, and cyclin D, with 44.8 positive cases. Selected photomicrographs of positive cases for each biomarker and control cases for MUC 1 are displayed in Figures 1 and 2. Based on the expression of ER, PR, HER2, and Ki-67, the cases were categorized into the breast cancer molecular subtypes. The triple-negative molecular subtype formed the largest group, constituting 54.3% of cases.

### 3.2. Statistical Analysis

The chi-squared test was computed for association between MUC1 and the molecular subtypes, other markers, and the clinicopathological characteristics.  Table 4 shows the percentages of cases of each parameter or marker which were positive for MUC1 expression, along with the chi-squared values and associated *p* values. Significant associations were observed between MUC1 overexpression and cyclin D (*p* < 0.001), HER2 overexpression (*p*=0.01), and triple negative (*p* < 0.05).

## 4. Discussion

This study sought to assess the predictive and prognostic significance of MUC1 in breast cancer presented by Ghanaian women in Kumasi. Various such studies have been conducted on breast cancer samples from the developed world, where it is overexpressed in a very high proportion of cases and is generally associated with good prognosis. Studies on MUC1 in African breast cancer samples are virtually nonexistent, as only a handful has been done. This is one of the first such studies in Ghana and hence provides novel information about the biology of breast cancer here, and by extension, West Africa.

The results indicate that the larger proportion of cases analysed overexpressed MUC1. This is in line with the long-held assertion that MUC1 is widely expressed in all forms of adenocarcinoma [23]. The proportion of MUC1 positives (59.0%) observed in the current study is however lower, compared to most Western studies, by stressing differences between breast cancer in African and Caucasians. Proportions of MUC1-positive cases reported by Western studies range from 86.4% [35] to 100% [30]. It is however in consensus with the 58% reported by Patel et al. [39] in a cohort of Indian cases. Elseed et al. [40] recorded 72.5% MUC1-positives in a study on Sudanese cases, the only prior African study on the prognostic significance of MUC 1 accessible to us.

The association between MUC1 overexpression and age and histological diagnoses revealed no significant statistical values (*p*=0.810 and 0.533, respectively). Likewise, no significant associations were observed between MUC1 positivity and histological grade. It was however observed that MUC1-positive cases were predominantly of higher grades (Table 4). This contrasts the published literature on western cases, where MUC1 positivity is often associated with lower histological grades [32, 34–36]. Again, no significant association was observed between MUC1 overexpression and Ki-67, yet MUC1-overexpressing cases were predominantly of high Ki-67 indexes, suggesting higher mitotic activities in these tumours.

It was observed that MUC1 overexpression was strongly associated with aberrant expression of cyclin D (*p* < 0.001). Cyclin D is a cell-cycle protein involved in the progression through the G1 stage to the S phase. Its overexpression in cancers has been described to be associated with enhanced proliferation rates, poor prognosis, and decreased survival [41]. Liu et al. [42] demonstrated expression of aberrant glycosylated MUC1 and activity induced sustained expression and stability of cyclin D, thereby promoting proliferation. Our findings on the association with cyclin D overexpression supports a study by Van Der Vegt et al. [33], who found a similar significant association between cytoplasmic MUC1 expression and cyclin D, and consequently, an association with poor prognostic factors.

Relating MUC1 expression pattern of our cases to the molecular subtypes revealed significant associations with two poor prognostic phenotypes. MUC1 overexpression was significantly exhibited by cases which were of the HER2 overexpression phenotype (*χ*^2^ = 7.057, *p* = 0.01). Although HER2 overexpression tumours are generally sensitive to trastuzumab therapy, they are inherently aggressive, with high proliferative rates, high histological grades, and frequent lymph node metastasis [43, 44]. None of the previous Western studies which were reviewed reported a significant association between MUC1 expression and HER2 overexpression [33, 34, 36]. Elseed et al. [40] made no report on the association between MUC1 and HER2 expression, as their study focused only on ER status and the triple negative phenotype. Our study is therefore the first known to us to report such an association in a cohort of African cases.

The triple-negative subtype also demonstrated a significant association with MUC1 overexpression (*χ*^2^ = 4.345, *p* < 0.05). The triple-negative phenotype is the predominant type portrayed by African women [45–47] and is characterized by aggressive histopathology, with high histological grades, high rates of proliferation, lymph node involvement, and distant metastasis. Do et al. [36] found an inverse association, where MUC1 negativity was rather predominant among the triple-negative cases in their cohort, while other studies found no such an association [39]. The sole prior study on MUC1 among Africans [40] found no association, although MUC1 was widely overexpressed and the cases were predominantly triple negative. Their inability to identify an association may however be explained by the small sample size used, which could not afford the study the desired statistical significance.

## 5. Conclusion

MUC1 was seen to be overexpressed in more than half of the cases tested. These tumours overexpressing MUC1 generally had high histological grades and high Ki-67 and cyclin D expression and were frequently involved in lymph node metastasis. Significant associations were seen between MUC1 and the HER2 overexpression, as well as the triple-negative subtypes.

This study therefore implies that MUC1 overexpression in breast cancer in Kumasi predicts poor prognosis, with aggressive tumour types that may be highly associated with metastasis.

## Figures and Tables

**Table 1:** Details of antibodies used.

Antibody	Clone	Pretreat	Dilution	Control	Company	Address
CYCLIN-D1	SP4	ER2/20	1 : 40	88-13792-7A	THERMO SCIENTIFIC	Grandy Island, NY
ER	1D5	ER1/20	1 : 50	Breast CA	BioCare Medical	Concord, CA
HER-2		ER1/20	RTU	Breast CA	DAKO	Carpinteria, CA
Ki-67	MIB-1	ER1/20	1 : 80	Tonsil	DAKO	Carpinteria, CA
MUC-1	Ma695		1 : 160	Colon CA	Leica Microsystems	Buffallo Grove, IL
PR	PgR 636	ER1/10	1 : 400	Endo/Myome	DAKO	Carpinteria, CA

**Table 2:** Descriptive statistics of TMA cases.

Age distribution	Mean/years	Standard deviation/years
	49.34	13.739

Histological diagnoses	Type	Frequency (percentage)
	Invasive carcinoma NST	166 (83.0)
	Ductal carcinoma in situ	9 (4.5)
	Metaplastic carcinoma	6 (3.0)
	Invasive lobular carcinoma	5 (2.5)
	Mucinous carcinoma	5 (2.5)
	Invasive papillary carcinoma	2 (1.0)
	Medullary carcinoma	2 (1.0)
	Others	5 (2.5)

Histological grade	Grade I	14 (9.4)
	Grade II	52 (34.9)
	Grade III	83 (55.7)

Lymphovascular invasion	Positive	32 (49.2)
	Negative	33 (50.8)

**Table 3:** Immunohistochemical characteristics of the cases.

Biomarker	Frequency	Percent
ER		
Positive	54	29.0
Negative	132	71.0

PR		
Positive	20	10.9
Negative	163	89.1

HER2		
Positive	37	20.7
Negative	142	79.3

MUC1		
Positive	102	59.0
Negative	71	41.0

Ki-67		
Low	113	62.1
Moderate	30	16.5
High	39	21.4

Cyclin D		
Positive	81	44.8
Negative	100	55.2

Molecular subtype		
Luminal A	32	19.8
Luminal B	16	9.9
HER2 overexpression	26	16.0
Triple negative	88	54.4

**Table 4:** Statistical analysis of MUC1 expression in relation to other parameters.

	MUC1 positive (%)	Chi-squared	*P* value
Clinicopathological characteristics			
Age		38.412	0.810
Histological diagnosis		8.993	0.533
Histological grade			
I	53.8	0.236	0.889
II	61.4		
III	59.4		
Lymphovascular invasion	63.0	0.639	0.575
Molecular subtypes			
Luminal A	60.7	0.122	0.828
Luminal B	68.8	0.207	0.787
HER2 overexpression	87.5	**7.053**	**0.01**
Triple negative	56.6	**4.345**	**<0.05**
Other markers			
Cyclin D	75.3	**16.345**	**<0.001**
Ki-67			
Low	53.5	5.652	0.059
Moderate	69.0		
High	73.7		

## Data Availability

The excel data used to support the findings of this study may be released upon application to the Committee on Human Research, Publication and Ethics of School of Medical Sciences/Komfo Anokye Teaching Hospital, Block J, School of Medical Sciences, Kwame Nkrumah University of Science and Technology, Kumasi, Ghana
